# Accuracy of tumor segmentation from multi-parametric prostate MRI and ^18^F-choline PET/CT for focal prostate cancer therapy applications

**DOI:** 10.1186/s13550-018-0377-5

**Published:** 2018-03-27

**Authors:** Morand Piert, Prasad R. Shankar, Jeffrey Montgomery, Lakshmi Priya Kunju, Virginia Rogers, Javed Siddiqui, Thekkelnaycke Rajendiran, Jason Hearn, Arvin George, Xia Shao, Matthew S. Davenport

**Affiliations:** 1 Radiology Department, University of Michigan, Ann Arbor, MI USA; 2 Urology Department, University of Michigan, Ann Arbor, MI USA; 3 Pathology Department, University of Michigan, Ann Arbor, MI USA; 4 Department of Radiation Oncology, University of Michigan, Ann Arbor, MI USA; 5 Department of Radiology, Division of Nuclear Medicine, University of Michigan Health System, University Hospital B1G505C, 1500 E. Medical Center Drive, Ann Arbor, MI 48109-0028 USA

**Keywords:** Prostate cancer, Tumor segmentation, MRI, ^18^F-fluoromethylcholine, PET/CT, Focal therapy

## Abstract

**Background:**

The study aims to assess the accuracy of multi-parametric prostate MRI (mpMRI) and ^18^F-choline PET/CT in tumor segmentation for clinically significant prostate cancer. ^18^F-choline PET/CT and 3 T mpMRI were performed in 10 prospective subjects prior to prostatectomy. All subjects had a single biopsy-confirmed focus of Gleason ≥ 3+4 cancer. Two radiologists (readers 1 and 2) determined tumor boundaries based on in vivo mpMRI sequences, with clinical and pathologic data available. ^18^F-choline PET data were co-registered to T2-weighted 3D sequences and a semi-automatic segmentation routine was used to define tumor volumes. Registration of whole-mount surgical pathology to in vivo imaging was conducted utilizing two ex vivo prostate specimen MRIs, followed by gross sectioning of the specimens within a custom-made 3D-printed plastic mold. Overlap and similarity coefficients of manual segmentations (seg1, seg2) and ^18^F-choline-based segmented lesions (seg3) were compared to the pathologic reference standard.

**Results:**

All segmentation methods greatly underestimated the true tumor volumes. Human readers (seg1, seg2) and the PET-based segmentation (seg3) underestimated an average of 79, 80, and 58% of the tumor volumes, respectively. Combining segmentation volumes (union of seg1, seg2, seg3 = seg4) decreased the mean underestimated tumor volume to 42% of the true tumor volume. When using the combined segmentation with 5 mm contour expansion, the mean underestimated tumor volume was significantly reduced to 0.03 ± 0.05 mL (2.04 ± 2.84%). Substantial safety margins up to 11–15 mm were needed to include all tumors when the initial segmentation boundaries were drawn by human readers or the semi-automated ^18^F-choline segmentation tool. Combining MR-based human segmentations with the metabolic information based on ^18^F-choline PET reduced the necessary safety margin to a maximum of 9 mm to cover all tumors entirely.

**Conclusions:**

To improve the outcome of focal therapies for significant prostate cancer, it is imperative to recognize the full extent of the underestimation of tumor volumes by mpMRI. Combining metabolic information from ^18^F-choline with MRI-based segmentation can improve tumor coverage. However, this approach requires confirmation in further clinical studies.

## Background

The rationale for focal therapy of prostate cancer is based on the concept of minimizing damage to off-target tissues while achieving successful local treatment response. Focal treatment methods include cryotherapy, laser ablation, high-frequency focused ultrasound (HIFU), irreversible electroporation, and focal brachytherapy [1–4]. The benefit compared to radical treatment is reduced invasiveness that lessens patient morbidity. The key to the success of focal therapies is appropriate patient selection based on an unequivocal, usually solitary, treatment target [5].

For focal therapies, it is imperative not only to accurately differentiate significant from indolent prostate cancer [6, 7], but also to precisely identify the tumor borders. In recent years, multi-parametric prostate MRI (mpMRI) has emerged as a useful tool for the detection and risk stratification of primary prostate cancer and recently has been incorporated into American Urological Association (AUA) algorithms for patients with suspected or low-risk prostate cancer [8–10]. When used optimally, mpMRI can cost-effectively identify a significantly greater fraction of clinically significant cancers (Gleason score ≥ 7 [≥ 3 + 4]) compared to standard biopsy alone while minimizing the detection of low-risk cancer [11–13]. These outcomes are important because accurate risk stratification of primary prostate cancer historically has been fraught with overdiagnosis and overtreatment [14–16]. However, mpMRI is not perfect. It suffers from a large false-positive rate [17, 18], only moderate inter-rater agreement [19], and a steep learning curve [20], each resulting in unnecessary biopsies that drive complication rates and unwanted detection of low-risk disease.

It has recently been demonstrated that the addition of positron emission tomography (PET) with ^18^F-choline to mpMRI (PET/MRI) can significantly improve the identification of Gleason ≥ 3+4 prostate cancer over mpMRI alone [21], mainly by improving on the comparatively low specificity of mpMRI [17]. The purpose of this study was to determine the precision of tumor boundary detection (segmentation) by visual inspection of mpMRI and by semi-automatic segmentation based on ^18^F-choline PET in patients undergoing prostatectomy. Due to prostate deformations, differing slice thicknesses between imaging and histology, and differing spatial orientation between imaging and whole-mount pathology, registration of medical imaging to pathology from prostatectomy specimen is challenging [22]. To overcome these issues, we first accurately registered in vivo imaging to the prostatectomy specimen using a multi-step approach presented in this paper.

## Methods

### Patient population

We report on a subset of patients within a Health Insurance Portability and Accountability Act-compliant registered prospective trial (Clinicaltrials.gov identifier: NCT01751737) to assess the value of mpMRI and ^18^F-choline PET prior to targeted and standard prostate biopsies [21]. The institutional ethics committee approved this protocol. Written informed consent was obtained. Ten study subjects with rising PSA values and suspected or known untreated localized adenocarcinoma of the prostate recruited between November 2013 and June 2016 subsequently underwent prostatectomy following the detection of Gleason ≥ 3+4 cancer at biopsy. The majority of patients had additional low-grade (Gleason 3+3) cancers at final pathology (Table 1), but such low-grade disease was not the subject of this study.

**Table 1:** Patient characteristics

Patient	Age (years)	PSA at time of biopsy (ng/mL)	Time interval between MRI and PET (days)	Time interval between imaging and biopsy (days)	Time interval from biopsy to prostatectomy (days)	Volume of Gleason ≥ 7 disease at pathology (mL)	Standard biopsy highest Gleason score	Targeted biopsy highest Gleason score	Final maximum Gleason score at prostatectomy	Number of additional Gleason 3+3 cancers at prostatectomy
1	57	4.6	0	23	63	0.19	3 + 3	3 + 4	3 + 4	4
2	73	4.3	0	21	61	1.35	3 + 4	Negative	3 + 4	6
3	73	4.6	0	15	133	1.35	3 + 3	3 + 4	3 + 4	2
4	60	23.0	19	6	55	3.15	3 + 4	4 + 4	4 + 3	1
5**	64	3.0	0	14	120	0.15	3 + 3	3 + 3	3 + 4	4
6	65	24.4	25	56	99	1.37	Negative	3 + 4	4 + 3	0
7	62	4.6	47	28	146	0.57	Negative	4 + 3	4 + 3	2
8	66	20.4	45	49	106	6.31	3 + 4	4 + 3	4 + 3	0
9	59	4.7	37	35	169	0.91	3 + 3	4 + 5	4 + 5	0
10	66	8.6	35	7	79	3.73	Negative	3 + 4	3 + 4	0

**Excluded from analysis as lesion was not identified by readers

The study participants, 65 ± 5 years of age (range 57–73), had 1.2 ± 0.6 (range 0–2) biopsy procedures prior to entering the trial. Three subjects did not have a prior biopsy. Previous biopsies resulted in four Gleason 3+3 cancers, one Gleason 3+4 cancer, and no prostate cancer in the remaining two cases. PSA levels at study entry ranged from 3.0 to 24.4 ng/mL (mean 8.65 ± 8.06).

### Multi-parametric prostate MRI

All MR imaging was performed on the same 3 T unit (Ingenia, Philips Healthcare, Andover, MA, USA) without an endorectal coil using a 16-channel phased array coil. Technical details are provided in the literature [21]. Briefly, the following in vivo MRI pelvic examinations with and without contrast material were acquired prior to biopsy: axial 3D T2-weighted (T2w) fast spin echo (FSE) (voxel size 1.0 × 1.0 × 1.0 mm; repetition time 2051 ms; echo time (TE) 333 ms); axial/sagittal/coronal T2w 2D FSE (voxel size 0.7 × 0.9 × 3.0 mm; repetition time 4758 ms; TE 110 ms); axial diffusion-weighted imaging (voxel size 2.3 × 2.4 × 3.0 mm; b-factors 0, 100, and 800 s/mm^2^); and axial T1w pre- and dynamic post-contrast 3D spoiled gradient echo with spectral adiabatic inversion recovery fat saturation (dynamic contrast-enhanced (DCE); in-plane voxel size 0.9 × 0.9 mm). Patients were encouraged to empty their bladder prior to scanning to minimize deformation effects on the prostate. An apparent diffusion coefficient (ADC) map was reconstructed for all diffusion-weighted imaging sequences, and subtraction imaging was generated for all DCE sequences.

For ex vivo MRI, axial and coronal 3D T2w FSE (voxel size 0.75 × 0.75 × 0.75 mm; repetition time 2451 ms; TE 320 ms) and axial diffusion-weighted imaging (voxel size 0.5 × 0.5 × 3.0 mm; b-factors 0 and 800 s/mm2) were acquired.

### ^18^F-choline PET/CT

^18^F-choline was synthesized as described in the literature [23]. MRI and ^18^F-choline PET/CT were acquired separately on average 21 days apart (range 0–47 days). ^18^F-choline PET/CT scans were performed on a Biograph TrueV mCT scanner with extended field of view and a 40-channel helical CT (Siemens Medical Solutions, Malvern, PA, USA) with an intrinsic axial resolution of 4.1 mm FWHM and time-of-flight reconstruction [24]. Twenty minutes after IV injection of approximately 230 MBq of ^18^F-choline, a low-dose CT transmission scan and a 10-min emission scan of the lower abdomen and pelvis were performed. Images were reconstructed using established methods [21].

### Tumor segmentations

For segmentation of prostate cancer lesions, all mpMRI series were reviewed on a PACS workstation (McKesson, San Francisco, CA, USA). Two fellowship-trained expert prostate MRI readers were tasked to contour the histologically known significant prostate cancer on the T2w sequence simulating a target volume definition for focal prostate cancer treatment. For this purpose, the readers had full knowledge of the prior reports of the in vivo mpMRI scans as well as all histological reports of prior biopsies, including targeted biopsies based on mpMRI lesions identified. The readers were blinded to the segmentation of the other reader as well as blinded to the results from ^18^F-choline PET, the ex vivo specimen MRI scans, and the final histology from prostatectomy. After identification of a candidate lesion, an individual tumor volume of interest (VOI) was defined based on the basis of visual perception of the lesion borders on a MIM Maestro (MIM Software, Cleveland, OH, USA) workstation and assigned as seg1 (reader 1) and seg2 (reader 2) for each case. A semiautomatic gradient-based segmentation method (PETEdge, MIM Software, Cleveland, OH, USA) without manual adjustments was used to determine the borders of the MRI-identified lesions on ^18^F-choline PET (assigned as seg3) [25]. Furthermore, all three segmentation volumes of a given tumor were spatially combined (union of seg1, seg2, seg3 = seg4) for further analysis. Gradient edge detection identifies tumor on the basis of a change in SUV at the tumor border. The particular gradient method used calculates spatial derivatives along tumor radii then defines the tumor edge on the basis of derivative levels and continuity of the tumor edge. This well-established method has been shown in phantom studies to be more accurate and reproducible compared to manual segmentation as well as threshold methods determining the tumor border on the basis of a percentage of the maximum activity within the tumor [26].

We also determined the accuracy of volume estimates based on a commonly applied ellipsoid formula. For this purpose, the maximum distance of the tumor in three dimensions (transverse, coronal, sagittal) was determined and the tumor volume was calculated based on the ellipsoid formula (length × width × height × 0.52) [27]. This formula-based tumor volume estimate as well as all other segmentations (seg1, seg2, seg3, seg4) were compared to a true volumetric measurement obtained from histology as depicted on high-resolution 3D T2w MRI as standard of reference. Here, the tumor volume (in milliliters) is defined by the sum of voxels encompassed by the individually drawn tumor VOI. Accordingly, any part of the standard of reference (histological) tumor volume that was not included in the tumor volume estimate (segmentation or formula-based) is defined as the underestimated tumor volume.

### Pathological evaluation

Whole-mount sections after prostatectomy were processed for routine histological assessment (hematoxylin/eosin (HE) stain) using the paraffin embedding process and 3-μm sections. Each tumor focus was assigned a primary and secondary Gleason grade and staged according to the American Joint Committee on Cancer guidelines [28]. All tumor foci were microscopically assessed whether any inhomogeneity of Gleason pattern within Gleason ≥ 3+4 cancers was present. For this study, significant prostate cancer was defined conservatively as any Gleason ≥ 3+4 cancer regardless of the tumor volume [29].

### Registration process PET to MRI

First, PET data were registered onto 3D T2w MR using commercially available software (MIM Maestro, Cleveland, Ohio, USA). The result of rigid registrations was visually assessed using pelvic bones as landmarks. In selected cases when rectal content or bladder filling shifted the position of the prostate, a constrained intensity-based, free-form deformable registration was added to register the prostate on MRI and PET [30]. Such registration was possible using the outline of the bladder base and internal prostatic structures (such as BPH nodules) identified on both imaging as landmarks.

### Registration process histology from prostatectomy to MRI and PET

**Fig. 1:**
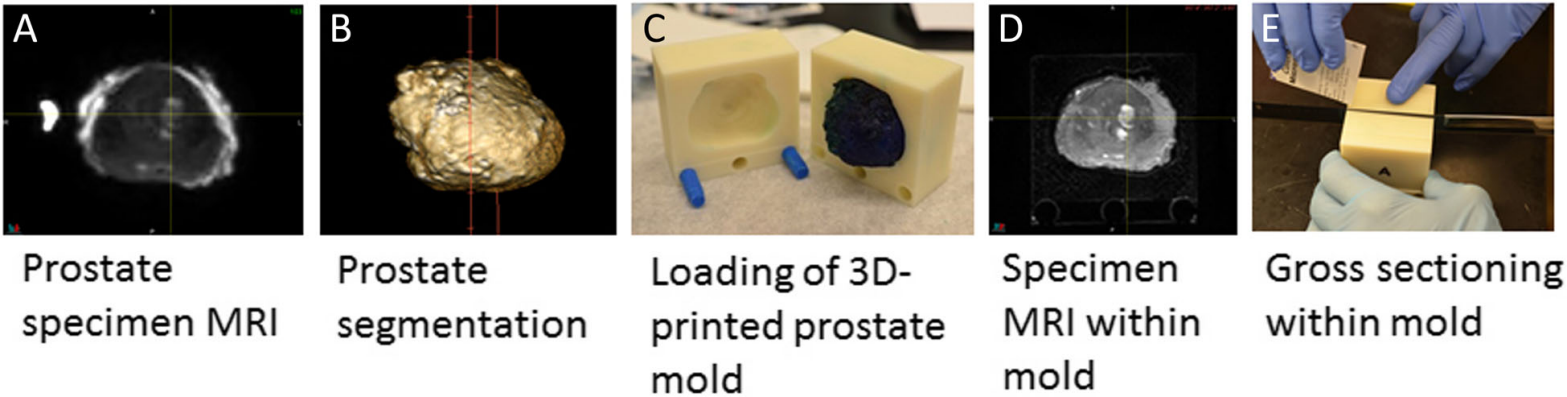
Registration process step 1. Following prostatectomy, a 3D T2W MRI scan of the prostate specimen was performed (**a**), to accurately segment the specimen for generating a mold design (**b**). After 3D printing, the specimen was placed into the mold (**c**), and a second high-resolution 3D T2W MR scan was performed (**d**). Then, the specimen was gross sectioned within the mold (**e**)

The second and more difficult registration step was the mapping of histology onto MRI through the use of the ex vivo prostate specimen MRI. Following prostatectomy, an ex vivo T2w 3D specimen MRI was performed with the prostate immersed in perfluorocarbon solution. This solution was used to avoid susceptibility artifacts (Fig. 1a) [21]. Using the specimen MRI data, the prostate was segmented on a 3D workstation (Vitrea, Vital Images, Minnetonka, MN, USA). The segmentation served as the basis for a 3D-printed mold (Fig. 1b) that could hold the prostate (Dimension Elite 3D, Stratasys, Eden Prairie, MN, USA). The mold design included slits spaced 3 mm apart that were later used to facilitate accurate gross sectioning of the prostate specimen within the mold (Fig. 1c). The specimen MRI also facilitated image registration utilizing intra-prostatic features (e.g., BPH nodules).

**Fig. 2:**
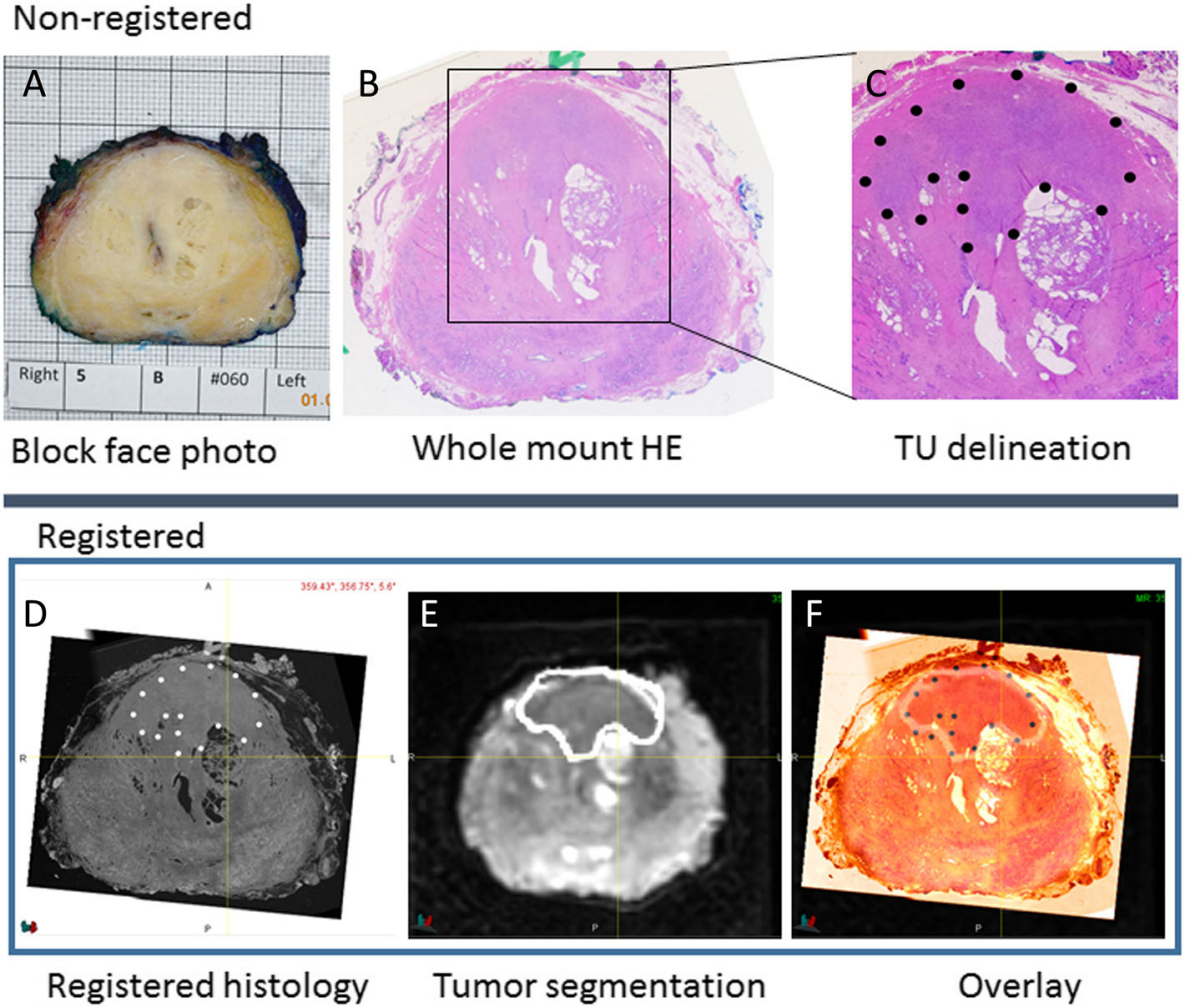
Registration process step 2. Individual sections of the specimen were photographed (**a**) and whole-mount sectioned for HE histology (**b**). The tumor borders were marked by the pathologist (**c**). Stacked histology slices (**d**) were registered to the mold MRI scan (**e**). Registration accuracy can be checked on overlay images (**f**)

Following complete fixation of the prostate, the prostate borders at the base and apex were gross sectioned off the specimen to allow detailed histological assessments for potential extraprostatic extension. Then, a second MR scan was performed with the prostate specimen positioned within the mold (Fig. 1d). Next, the prostate was gross sectioned without repositioning (Fig. 1e), linking corresponding histological whole-mount HE slides to their respective sectioning planes from the second ex vivo MRI. Since microtome sectioning for histology regularly deforms the HE slide relative to its representation on MR, we obtained block-face photographs at the time of gross sectioning (Fig. 2a). If notable deformation resulted from sectioning, we utilized these block-face photographs to register each deformed HE slide back to its original shape at the time of gross sectioning.

Volumetric 3-mm stacks of HE whole-mount histology sections (Fig. 2b) were then registered onto the ex vivo high-resolution specimen MRI, providing a consistent registration of the ex vivo MRI to pathology at 3-mm intervals. The registration of whole-mount HE and ex vivo HR MRI was rigid for the respective slice at the slit level. However, in between those intermittent “rigid” registrations, deformable registration was often required. Since a given HE slice is repeated to cover a 3-mm-thick space (in *z*-direction), deformable registration was needed to follow contour and intraprostatic changes seen on MRI. In essence, the prostate borders and well-identifiable intraprostatic structures (urethra, BPH nodules, tissue borders between peripheral and transitional zones) were aligned using constrained intensity-based, free-form deformable registration.

**Fig. 3:**
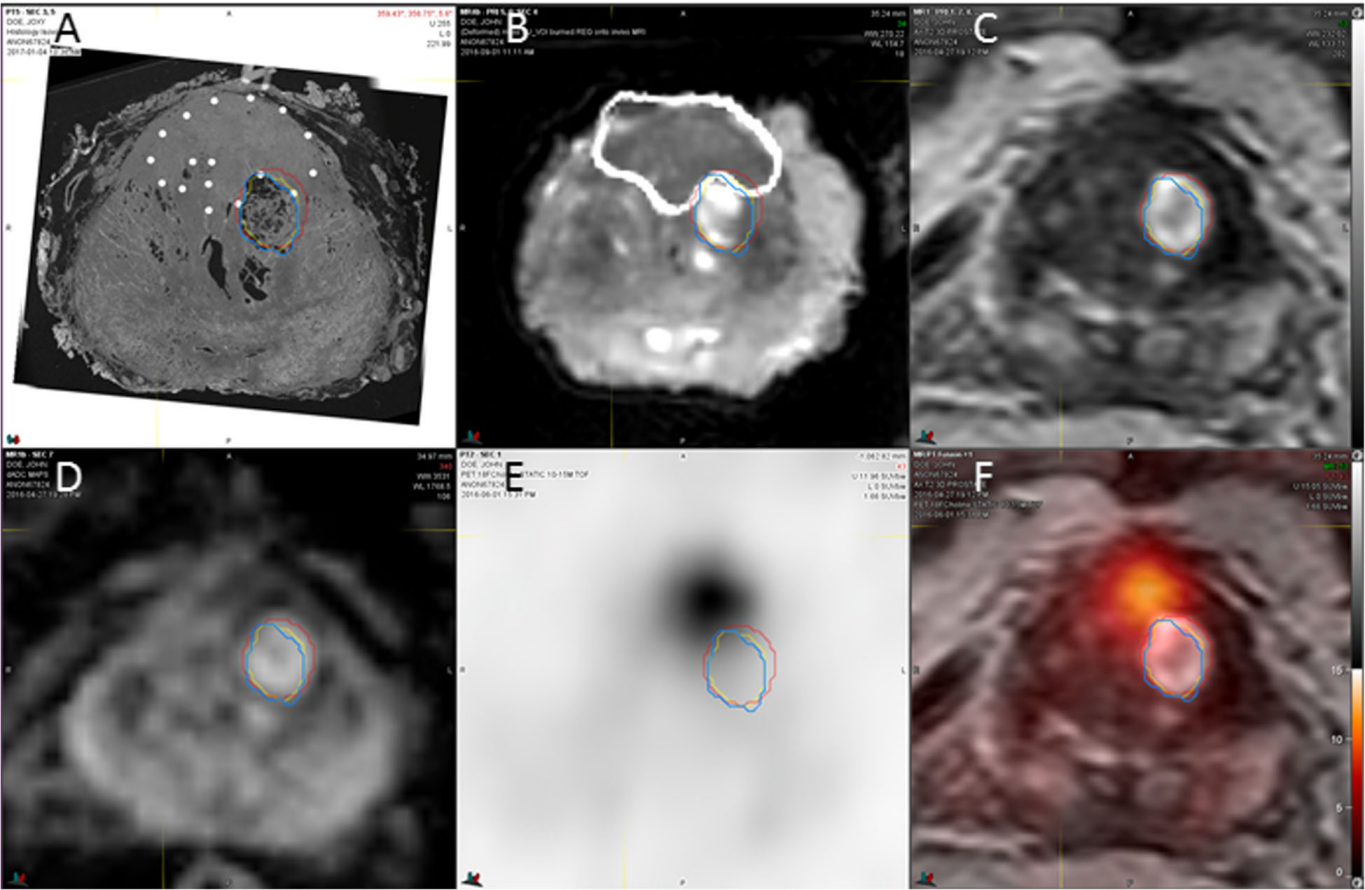
Determination of registration accuracy. Registered transaxial HE histology (**a**), specimen 3D T2W MRI (**b**), in vivo 3D 2TW MRI (**c**), ADC (**d**), ^18^F-choline PET (**e**), and fusion PET/MRI (**f**) are shown (SUV range 0–15). Contours of a BPH nodule obtained from histology (blue), ex vivo mold MRI (yellow), and in vivo MRI (red) are displayed on corresponding slices. Also, contours of a Gleason 3+4 adenocarcinoma in the anterior gland are shown on histology (**a**) and ex vivo corresponding specimen MRI (**b**)

All prostate cancer foci were outlined by the pathologist on whole-mount HE slices (Fig. 2c), and lesion boundaries were transferred into the ex vivo MRI (Fig. 2d/e). The registration quality was checked on overlay images (Fig. 2f), and the ex vivo T2w 3D MRI with embedded histological information was registered back into the in vivo T2w 3D MRI (Fig. 3).

### Assessment of registration accuracy

We determined the accuracy of image registrations between in vivo T2w MR and histology using well-delineated BPH nodules as landmarks. One suitable BPH nodule was identified in each of 10 prostatectomy cases. First, the respective BPH nodules were segmented on stacked HE histology slices. Since HE slices were not continuous, but 3 mm apart, we used software interpolation between each slice to fill the resulting gaps. Then, the same BPH nodules were individually segmented on ex vivo and in vivo MRI volumes (Fig. 3). Common similarity coefficients were calculated to determine registration accuracy: Hausdorff distance (HD), mean distance to agreement (MDA), and Dice and Jaccard [31]. Dice and Jaccard coefficients are similar statistical measures of the spatial overlap between two volumes as follows:

1 $$ \mathrm{Dice}\left(A,B\right)=\frac{2\ \left|A\right.\cap \left.B\right|}{\left|A\right|+\left|B\right|}, $$

2 $$ \mathrm{Jaccard}\left(A,B\right)=\frac{\left|A\cap \left.B\right|\right.}{\left|A\cup B\right|}, $$

where *A* denotes the tumor volume estimate and *B* the standard of truth (histological) tumor volume. Here, Dice is defined as 2 × intersection volume/total sum of volumes, while Jaccard describes the volume of intersection between two volumes/volume of the union of these volumes. Both overlap coefficients normalize the degree of intersection from 0 (no overlap) to 1 (perfect overlap). The spatial distance coefficients HD and MDA describe the volumetric maximum (HD) and mean distances (MDA) across all points on a surface and their closest point on another surface, where 0 mm reflects ideal outcome.

### Statistics

Statistical analyses were performed using JMP 13 (SAS, Cary, NC). Data were not normally distributed. To compare contour measurements (seg1–3 vs. histological truth), a paired Wilcoxon signed-rank test was performed. To compare similarity coefficients among readers (seg1,2) vs. PET-based segmentation routine (seg3), an unpaired Wilcoxon test was used. Data are presented as mean ± standard deviation (SD), and *P* values less than 0.05 were considered statistically significant.

## Results

Patient characteristics are summarized in Table 1. Histological tumor volumes were small with a median of 1.37 mL (range 0.15 to 6.31 mL). The Gleason ≥ 3+4 tumor with the lowest volume (case 5 in Table 1) was not identified by both readers and excluded from further analysis. The median time between mpMRI and PET, and the median time between the second MRI scan and the biopsy procedure were both 22 days. The median time from biopsy to prostatectomy was 102 days (range 55–169 days). The histological results from standard biopsies were upgraded on targeted biopsies in 8 of 10 cases. In one case, the targeted biopsy missed a Gleason 3+4 index cancer (case 2) identified on standard biopsy. No significant intratumoral variations in Gleason pattern within Gleason ≥ 3+4 cancers were observed.

Figure 3 demonstrates the accuracy of BPH nodule registrations obtained from pathology, ex vivo MRI, and in vivo MRI. Overall registration accuracy between in vivo MRI and histology was excellent with maximum HD below 2.7 mm and mean MDA below 0.5 mm with limited inter-individual variability as indicated by their respective standard deviations (Table 2). The overlap coefficients Dice and Jaccard were high between in vivo MRI and final pathology. Note that the final registration distance errors (in HD and MDA) between in vivo MRI and histology were less than the sum of their respective uncertainties for each registration step (Table 2).

**Table 2:** Accuracy of BPH nodule registration across mpMRI and histology

*N* = 10	Volume (in mL)	HD (in mm)	MDA (in mm)	Dice	Jaccard
Histology vs. ex vivo T2w MRI	2.84 ± 3.56 [0.85; 0.15–10.23]	1.64 ± 0.51 [1.47; 0.9–2.5]	0.28 ± 0.09 [0.29; 0.14–0.41]	0.90 ± 0.04 [0.91; 0.81–0.96]	0.83 ± 0.07 [0.83; 0.68–0.93]
Ex vivo T2w MRI vs. in vivo T2w MRI	2.99 ± 3.72 [0.96; 0.2–11.01]	2.27 ± 0.71 [2.03; 1.66–3.99]	0.41 ± 0.12 [0.42; 0.21–0.63]	0.86 ± 0.07 [0.85; 0.76–0.97]	0.76 ± 0.11 [0.74; 0.62–0.94]
In vivo T2w MRI vs. histology	3.0 ± 3.75 [1.0; 0.15–11.18	2.54 ± 0.57 [2.53; 1.6–3.67]	0.45 ± 0.09 [0.43; 0.37–0.68]	0.85 ± 0.07 [0.86; 0.73–0.94]	0.74 ± 0.11 [0.75; 0.57–0.88]

Mean ± standard deviation [median; minimum–maximum range]

*HD* Hausdorff distance, *MDA* mean distance to agreement

**Fig. 4:**
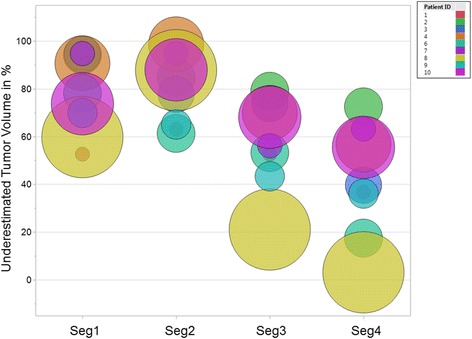
Bubble plot of underestimated tumor volumes. The percentage underestimation of the true tumor volume as determined by histology is given for the two human readers (seg1, seg2), the gradient-based segmentation method (seg3), and the union of seg1–3 (seg4). The diameter of the colored bubbles is proportional to the true tumor volume, and the color reflects the patient ID across segmentations (see Table 1). Please note that the underestimated tumor volume for the patient with the longest time period between imaging and prostatectomy, harboring the only high-risk cancer in this cohort (patient ID 9), is well within the range of the remaining patients

**Fig. 5:**
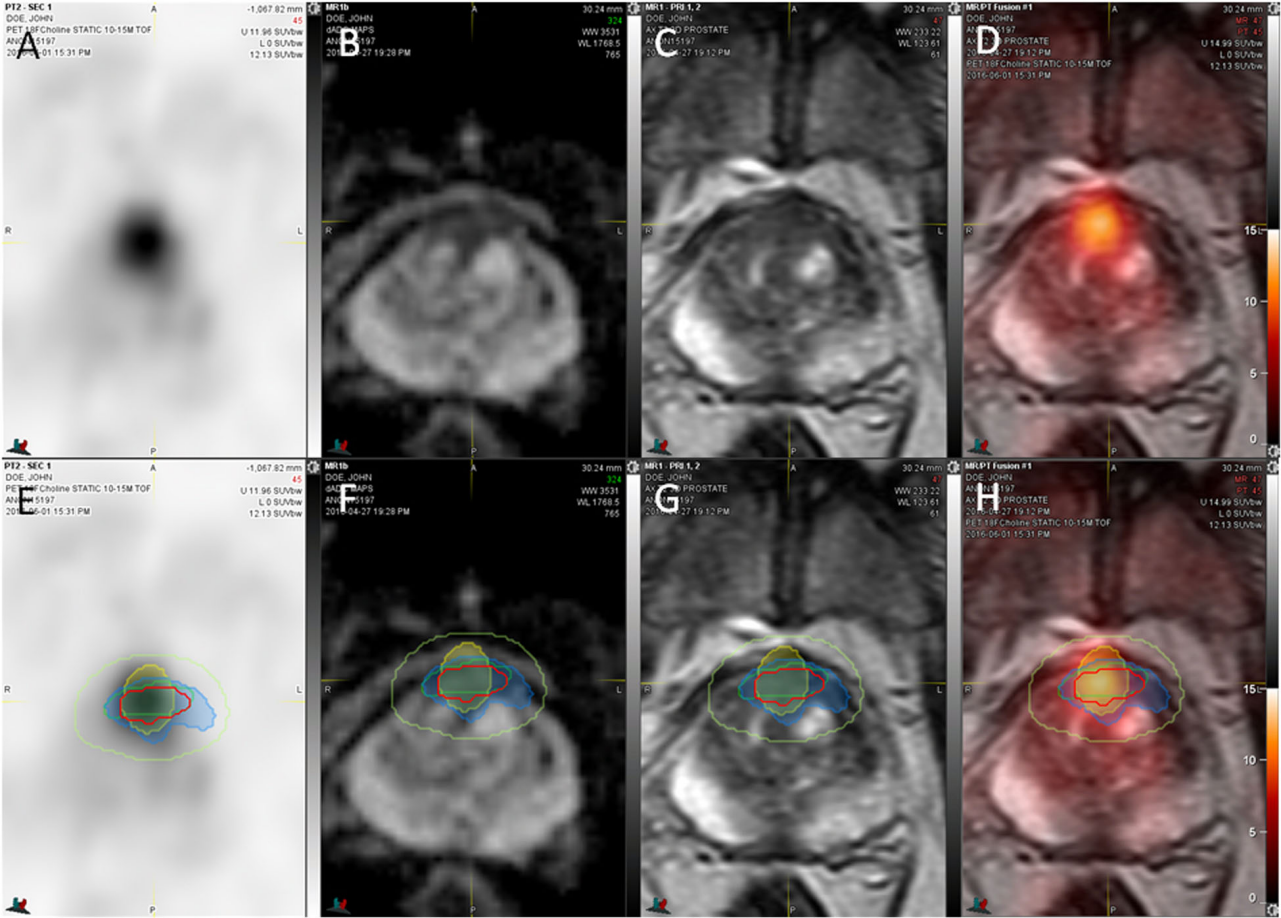
Tumor segmentation results. Registered transaxial ^18^F-choline PET (**a**), ADC (**b**), T2W MRI (**c**), and ^18^F-choline fusion PET/MRI (**d**) are shown (SUV range 0–15). Contours of a Gleason 3+4 prostate cancer are displayed on corresponding slices obtained from registered histology (blue line and contour shadow) and compared to registrations from two human readers (red and green line and a contour obtained from ^8^F-choline PET (yellow)) using a semiautomatic thresholding method (**e**–**h**). A contour combining these three segmentations with an added safety margin of 5 mm (lime line) completely covers the histological tumor volume

The results of tumor border segmentations relative to the histological reference standard are listed in Table 3. Visual tumor border segmentation based on MRI and PET-based thresholding substantially underestimated the true tumor volumes (seg1 by 79%, seg2 by 80%, seg3 by 58%). As seen from Fig. 4, regardless of the segmentation method, the true tumor volume has no major impact on the level of underestimation. Also, the underestimated tumor volume of the single high-risk (Gleason 4+5) cancer found (patient 9) is among the range of otherwise intermediate risk cancers (Table 1). Imaging findings of a typical example case are shown in Fig. 5. Similarity coefficients obtained from MRI and PET contours reveal misplaced tumor borders compared to histology (Table 3). Applying an ellipsoid formula to estimate the VOI volume resulted in a significant overestimation of the segmented volume by an average 48, 46, and 29% for seg1–3, respectively (*p* < 0.004 for each) (Table 3).

**Table 3:** Agreement of manual and semi-automated tumor segmentation with histology

*N* = 9	Reference histological volume (mL)	Ellipsoid-based tumor volume estimate (mL)	Segmented tumor volume (mL)	Underestimated tumor volume (mL)	Underestimated tumor volume (mL) in % of histological volume	HD (mm)	MDA (mm)	Dice	Jaccard
Reader 1 (seg1)	2.06 ± 1.95 [1.37; 0.19–6.31]	1.14 ± 1.26 [0.69; 0.08–3.97]	0.59 ± 0.79 [0.27; 0.07–2.53]	1.55 ± 1.22 [1.19; 0.12–3.77]	78.7 ± 15.9 [78.1; 52.6–94.7]	9.33 ± 3.84 [8.83; 2.2–15.7]	2.79 ± 1.51 [2.37; 0.56–5.5]	0.35 ± 0.20 [0.44; 0.09–0.61]	0.22 ± 0.14 [0.28; 0.05–0.44]
Reader 2 (seg2)		0.93 ± 0.58 [0.86; 0.23–1.96]	0.50 ± 0.36 [0.32; 0.15–1.17]	1.82 ± 1.75 [1.17; 0.2–5.54]	80.1 ± 14.0 [84.78; 61.3–98.6]	10.69 ± 4.12 [11.5;3.7–15.8]	3.15 ± 1.50 [3.15; 1.0–4.95]	0.30 ± 0.15 [0.28; 0.07–0.53]	0.19 ± 0.11 [0.16; 0.03–0.36]
^18^F-Choline PETEdge (seg3)		2.36 ± 4.19 [1.09; 0.28–13.39]	1.67 ± 3.07 [0.84; 0.15–9.75]	1.05 ± 0.80 [0.93; 0.16–2.54]	57.5 ± 17.9 [56.1; 21.1–79.0]	8.86 ± 2.72 [9.28; 3.6–12.5]	2.12 ± 0.63 [2.27; 0.93–3.11]	0.49 ± 0.11 [0.51; 0.32–0.63]	0.33 ± 0.09 [0.35; 0.19–0.46]
Combined (seg4)		N/A	2.28 ± 3.03 [1.55; 0.26–10.17]	0.71 ± 0.71 [0.36; 0.07–2.07]	42.4 ± 22.1 [39.7; 3.17–72.5] *p = 0.004 ^#^*P* = 0.002	8.11 ± 2.67 [8.64; 3.6–12.0]	1.84 ± 0.72 [1.75; 0.78–3.0]	0.52 ± 0.13 [0.56; 0.3–0.66]	0.36 ± 0.11 [0.39; 0.18–0.49] *p = 0.05 ^#^*P* = 0.005

Mean ± standard deviation [median; minimum–maximum range]

*VOI* volume of interest, *HD* Hausdorff distance, *MDA* mean distance to agreement, *N/A* not applicable

*Combined (seg4) vs. reader 1 (seg1)

^#^Combined (seg4) vs. reader 2 (seg2)

When a 5-mm contour expansion was applied in all directions from the tumor border segmentation edge, the underestimated tumor volume decreased significantly compared to the original segmentation (seg1: from 1.55 to 0.27 mL; seg2: from 1.82 to 0.57 mL; seg3: from 1.05 to 0.15 mL, *P* < 0.001). However, due to variations in tumor shape and lack of centering of the original segmentation, minimum contour expansions of 15 mm (seg1–2) and 11 mm (seg3) would have been needed to fully cover all Gleason ≥ 7 disease.

As indicated by Dice coefficients ≤ 0.41 and Jaccard ≤ 0.27, agreement between human readers and human vs. PET-based segmentations was low (Table 4). In fact, human readers and PET-based segmentation often identified different regions of the histological tumor volume, which lead to an improvement of the segmentation accuracy when all segmentations were combined (seg4). The underestimated tumor volume of the combined segmentation was significantly smaller than that of the individual human readers (Table 3).

**Table 4:** Inter-reader agreement

Comparison	HD (mm)	MDA (mm)	Dice (range 0–1)	Jaccard (range 0–1)
Reader 1 (seg1) vs. reader 2 (seg2)	8.56 ± 4.80 [7.09; 2.84–16.15]	2.43 ± 2.42 [1.46; 0.73–8.42]	0.41 ± 0.19 [0.45; 0.0–0.62]	0.27 ± 0.13 [0.29; 0.0–0.44]
Reader 1 (seg1) vs. ^18^F-choline PETEdge (seg3)	8.25 ± 3.95 [8.30; 4.37–14.27]	2.47 ± 1.43 [2.08; 1.07–4.60]	0.37 ± 0.14 [.0.41; 0.11–0.50]	0.23 ± 0.10 [0.26; 0.06–0.34]
Reader 2 (seg2) vs. ^18^F-choline PETEdge (seg3)	9.73 ± 5.22 [8.72; 3.09–18.16]	2.97 ± 1.91 [2.11; 0.90–7.12]	0.32 ± 0.14 [0.27; 0.15–0.54]	0.20 ± 0.10 [0.16; 0.08–0.37]

Mean ± standard deviation [median; minimum–maximum range]

*HD* Hausdorff distance, *MDA* mean distance to agreement

As expected, including a safety margin around the segmentation volumes significantly decreased the underestimated tumor volume (Table 5). The combined segmentation volume (seg4) with a 5-mm safety margin covered the entire tumor volume in five of nine cases; in the remaining four cases, a missed tumor volume of 0.03 ± 0.05 mL (2.04 ± 2.84% of the total tumor volume) resulted (Fig. 5e–h). While the percent of the underestimated volume was small, a contour expansion up to 9 mm was required to cover the entire tumor of irregularly shaped lesions. In contrast, with even larger target volumes at the 10-mm expansion level, complete tumor coverage could not be achieved for all tumors in the study by either human reader or the PET-based routine alone.

**Table 5:** Effect of safety margins on tumor coverage

*N* = 9	Volume of tumor segmentation with a 5-mm safety margin (mL)	Underestimated volume of tumor despite a 5-mm safety margin (mL)	Percentage of underestimated tumor volume despite a 5-mm safety margin (%)	Volume of tumor segmentation with a 10-mm safety margin (mL)	Underestimated volume of tumor despite a 10-mm safety margin (mL)	Percentage of underestimated tumor volume despite a 10-mm safety margin (%)	Minimum necessary safety margin to ensure complete coverage (mm)
Reader 1 (seg1)	4.83 ± 3.97 [3.48; 1.81–13.7]	0.27 ± 0.24 [0.3; 0.0–0.6]	14.6 ± 15.7 [8.4; 0.0–47.6]	11.01 ± 6.33 [9.61; 5.43–24.94]	0.11 ± 0.32 [0.0; 0.0–0.95]	1.91 ± 4.74 [0.0; 0.0–14.29]	8.78 ± 3.63 [9.0; 2.0–15.0]
Reader 2 (seg2)	4.55 ± 1.72 [5.15; 1.66–6.99]	0.57 ± 0.72 [0.2; 0.0–1.8]	19.2 ± 15.9 [19.6; 0.0–49.1]	10.67 ± 3.19 [11.33; 5.09–15.95]	0.13 ± 0.25 [0.01; 0.0–0.77]	3.60 ± 6.74 [0.72; 0.0–20.64]	9.67 ± 3.71 [11.0; 3.0–15.0]
^18^F-Choline PETEdge (seg3)	7.42 ± 6.62 [6.06; 1.94–24.15]	0.15 ± 0.19 [0.02; 0.0–0.48]	7.61 ± 10.2 [3.5; 0.0–31.2]	14.27 ± 8.40 [12.47; 5.46–34.38]	0.004 ± 0.013 [0.0; 0.0–0.04]	0.15 ± 0.46 [0.0; 0.0–1.38]	7.33 ± 2.78 [7.0; 3.0–11.0]
Combined (seg4)	8.88 ± 6.50 [7.1; 2.44–24.83]	0.03 ± 0.05 [0.02; 0.0–0.14]	2.04 ± 2.84 [0.54; 0.0–8.0] *P = 0.03 ^#^ *P* = 0.01	16.07 ± 8.06 [14.17; 6.58–35.35]	0.0 ± 0.0 [0.0; 0.0–0.0]	0.0 ± 0.0 [0.0; 0.0–0.0]	6.00 ± 1.87 [5.0; 3.0–9.0]

Mean ± standard deviation [median; minimum–maximum range]

*VOI* volume of interest

*Combined vs. reader 1

^#^Combined vs. reader 2

## Discussion

The goal of focal prostate cancer therapies is to destroy the entire tumor lesion using a non-invasive, well-tolerated treatment. Such a treatment would preserve normal genitourinary function while providing sufficient therapeutic efficacy. In order to be effective, focal prostate cancer therapies require accurate localization of the disease. The high anatomic detail provided by mpMRI at 3 T appears well suited to provide necessary guidance. However, it has already been recognized that lesion extension is typically underestimated by mpMRI [32]. Therefore, all focal treatment approaches are performed with a safety margin [33]. The key question is how to optimally define such safety margins.

We developed and applied a methodology for objective retrospective registration of whole-mount pathology to in vivo MRI and PET imaging. This method enabled us to determine whether in vivo imaging correctly identified and classified all tumor lesions found at final pathology from prostatectomy specimen [21]. A similar approach was used to determine boundary errors based on visual image inspection on MRI as well as mathematical thresholding techniques on ^11^C-choline PET [34]. To our knowledge, this is the first report on the accuracy of prostate cancer segmentations from mpMRI and ^18^F-choline PET using registered whole-mount histology as the reference standard. For this performance test, we simulated the clinical situation of a radiologist tasked to delineate tumor borders prior to a hypothetical planned ablation, in which readers were aware of the interpretation of the mpMRI and biopsy-based histology but had no knowledge of the ^18^F-choline PET results, ex vivo MRIs, or the pathology from the prostatectomy specimen.

Determining the accuracy of prostate cancer segmentation in MRI is difficult. Typically, error metrics are derived from measured inter-observer variability [35]. This is subject to bias because it lacks a reference standard. We took a different approach by registering imaging to histology, thereby enabling error measurements of tumor border delineation. Our results indicated a substantial difference between “perceived” (i.e., by imaging) and “true” (i.e., by histology) tumor borders. Human expert prostate MRI readers and the PET-based routine were equally unsatisfactory. Depending on tumor shape and the identification of the lesions’ center by human readers, minimum contour expansions between 11 and 15 mm would have been needed to cover all Gleason ≥ 3+4 disease. Currently applied standard safety margins are typically 10 mm or less, which our results imply would often be insufficient.

Ouzzane et al. recently noted that only few studies with valid methodology attempted to address the crucial question of safety margins [4]. Depending on the planned focal treatment method and the lesion location within the prostate, 4–10 mm safety margins have been proposed [1, 36, 37]. For example, Ting et al. used irreversible electroporation to treat 25 men with low- to intermediate-risk prostate cancer with a 5-mm minimum safety margin. Short-term follow-up indicated a 24% recurrence rate at 8 months and almost all recurrences were adjacent to the treatment zone [2], suggesting that treatment of larger tumor volumes would have been needed. Safety margins of 4–6 mm near the sphincter muscle have been advocated to avoid incontinence. Ahmed et al. performed a prospective HIFU trial involving 56 men with low- (12%), intermediate- (84%), and high-risk (4%) prostate cancer. Fifty-two men received a 6-month post-treatment biopsy, and clinically significant cancer was found in 10 cases [38]. Other groups advocate hemiablation to achieve better long-term disease control with low morbidity [39]. Based on the available literature, it remains debatable whether focal prostate cancer treatment is a suitable alternative to established whole-gland treatment approaches.

While our registration method was designed to optimize the accuracy of registration between in vivo MRI and pathology, it is not without error. The quantification of registration errors is difficult because the processing of human prostate tissues has to follow established methodological standards. Since the bladder surface and the prostatic apex are undergoing separate histological assessments to evaluate for possible extracapsular extension of cancer, they are not part of the final prostate specimen. Fiducial markers could not be introduced into the specimen after prostatectomy because they would be dislodged during sectioning of the whole-mount blocks. Due to the stability constraints of plastic molds, slits for gross sectioning of the prostate within the mold were spaced at 3-mm intervals. Therefore, there was a maximum uncertainty of 3 mm and a mean uncertainty of 1.5 mm with respect to the presence or absence of a given lesion on consecutive slices. Using software interpolation between registered whole-mount HE slices, as done in this study, will however lessen uncertainties in tumor border definition. Deformations of the specimen relative to the in vivo geometry of the prostate are often non-uniform. Bending and warping energies are particularly high at the lateral peripheral zones of the prostate [22], which increases registration uncertainties at these locations. While registration errors of each registration step may add up, it is unlikely that they are always to the disadvantage of the lesion representation on in vivo T2W MRI used for tumor segmentation. In fact, when determining the registration errors of BPH nodules from in vivo over ex vivo MRI to histology, the HD and MDA of the final registration (in vivo MRI to histology) increased but was far less than the sum of the individual registration errors. Registration errors due to dehydration of internal prostate tissue components during fixation are unavoidable and will cause shrinkage of the prostate specimen compared to the prostate in the human body [22]. Schned et al. estimated that the in vivo tumor volume was on average 12.4% higher than that measured from prostatectomy pathology [40]. Our own measurements obtained from well-delineated BPH nodules agree with this assessment. The less-than-perfect overlap coefficients (Dice and Jaccard < 1) obtained from BPH nodules can therefore in part be attributed to fixation-induced BPH shrinkage. Conversely, tumor shrinkage may have been smaller than in normal glandular tissue due to higher cellularity of tumors. Nonetheless, tumor shrinkage bears the potential to further underestimate tumor volumes on imaging relative to pathology.

As recently reviewed, the data on tumor volume estimates by MRI relative to histology are mixed; however, most published data report an underestimation of tumor volumes by mpMRI [32]. What may appear surprising in our study is the extent of the underestimation compared to prior reports. When considering the technical improvements of mpMRI over the last decade with the availability of 3 T MRI, one would expect improving accuracy in tumor border delineation. However, considering three prior reports comparing mpMRI at 3 T with whole-mount histology [27, 41, 42], two of which used 3D molds for sectioning, underestimation of tumor volumes between 7 and 80% were noted. The lowest underestimation was found by Turkbey et al. [27]; however, their study was limited due to tumor volume measurements based on an ellipsoid formula rather than a true volumetric measurement, which based on our data significantly overestimates lesion volumes. Also, gross sectioning of prostate specimen was performed manually in almost half of the subjects, which is technically extremely difficult to be done at 4-mm intervals thereby introducing errors [22]. Furthermore, they did not account for misplacement of tumor borders on histology relative to in vivo MRI as done in our study. Le Nobin et al. did not use 3D molds to facilitate registration of MRI and histology, nonetheless reporting on average 57% greater tumor volumes on histology vs. mpMRI evaluating retrospectively 46 lesions of overall smaller volume (mean 1.1 mL) compared to our cohort [42]. More recently, Priester et al. studied 114 subjects undergoing prostatectomy using individually 3D-printed molds for sectioning. The mean volume (2.5 mL) of 107 clinically significant prostate cancers was similar to our data, and the majority of tumors were graded as Gleason ≥ 3+4 [41]. Although they did not determine volumetric similarity coefficients, their overall results are comparable to our study. Eighty percent of the cancer volume from matched tumors was outside of the MR-defined tumor volume, a result that is very similar compared to our own data. The mean HD between histology and mpMRI of clinically significant tumors was 15.6 mm, which was even worse compared to our data (HD of 9.3–10.7 mm for human readers).

A potential reason for the observed disparities of tumor volume estimates obtained from in vivo imaging vs. histology from prostatectomy specimen could have been related to interim tumor growth, particularly as 5 of 10 patients underwent surgery roughly 5–7 months after imaging. While absolute volume growth rates of untreated Gleason ≥ 3+4 cancers are unknown, adenocarcinomas of the prostate are typically progressing slowly [43]. Supporting evidence for slow progression of intermediate risk prostate cancer comes from the observation arms of the Scandinavian Prostate Cancer Group Study Number 4 [44] and the Protect trial [45]. Indeed, 9 of 10 patients included in our cohort harbored intermediate-risk (Gleason 3+4 or 4 + 3) cancer. In one patient with high-risk cancer (Gleason 4+5) and the longest time interval (204 days) between imaging and prostatectomy (patient 9 in Table 1 and Fig. 4), the underestimated tumor volume was however well within the range of other segmentations. Therefore, we consider interval tumor growth not to be a significant contributing factor for the observed underestimation of the tumor volumes.

While the underestimation of tumor volumes on PET can be attributed to technical aspects of PET imaging including partial volume effects limiting image resolution [46], the reasons for underestimated tumor volumes on MRI are less clear. One possibility could be an inhomogeneity of Gleason pattern within a given tumor, where the “detectable” center of such lesions has higher cellular density compared to the periphery of lesions. Since Gleason 3+3 cancers are often missed on MRI [41] and lack focal elevated ^11^C-choline [46] and ^18^F-choline uptake on PET above background [21], we investigated whether mpMRI may only have identified certain pockets of more aggressive disease (Gleason ≥ 3+4), while missing areas of low-grade disease within inhomogeneous prostate cancers. However, a specific analysis of all Gleason ≥ 3+4 cancers did not identify any inhomogeneity of Gleason pattern within individual lesions that could explain the profound underestimation of tumor volumes. Nonetheless, missed tumors are often small and have lower grade than visible tumors on MRI. In addition, non-visible disease may display benign prostatic glandular tissue intermixed with carcinoma [47]. Not only the cellular density or its distribution, but also the cellular composition, particularly the amount of interstitial stromal space, has an effect on DWI and derived ADC maps [48]. Since DWI plays an important role for visual identification of prostate cancer on mpMRI, this phenomenon could explain—at least in part—the underestimation of tumor volumes and underlying uncertainties about tumor borders.

## Conclusions

Visual tumor segmentation based by mpMRI and semi-automatic segmentation based on ^18^F-choline uptake measures significantly underestimated the true volume of Gleason ≥ 3+4 prostate cancer, and substantial safety margins (up to 15 mm in our series) are required to include all diseases. Combining MR-based human segmentations with a semi-automated thresholding approach based on ^18^F-choline PET reduced the necessary safety margin to a maximum of 9 mm. While further work in a larger cohort is needed to validate these findings, this approach might contribute to improving the outcome of focal therapies of significant prostate cancer.
